# Using flexible regression models for calculating hospital’s production functions

**DOI:** 10.1186/s12913-020-05465-2

**Published:** 2020-07-10

**Authors:** Francisco Reyes-Santías, Octavio Cordova-Arevalo, Elena Rivo-Lopez

**Affiliations:** 1 Departamento de Organización de Empresas y Marketing, Universidad de Vigo. Facultad de Ciencias Empresarias e Turismo, As Lagoas, Campus Universitario s/n, 32004 Ourense, Spain; 2 PEMEX, Mexico City, Mexico

**Keywords:** Hospital, Production function, Cobb-Douglas, GAM, Regression

## Abstract

**Background:**

The relative lack of flexibility of parametric models has led to the development of nonparametric regression techniques based on the family of generalized additive models. However, despite the potential advantages of using Generalized Additive Model (GAM) in practice many models have, until now, not been sufficiently explored in health economics problems.

It could be interesting to calculate a new flexible hospital production function by means of a GAM including interactions and to compare it with the classic model Cobb-Douglas in the prediction of the behavior of productive factors.

**Method:**

The flexible model considered has been the AM including the beds-facultative interaction. The covariates “Hospital”, being a categorical variable and “Year” being a continuous variable, have also been included in the model. Based on the estimation of the model penalized thin plate splines will be used to represent smoothed functions. In this configuration, the smoothed parameters will be estimated via REML.

**Results:**

Cobb-douglas model fits well for the production functions of the more general clinical and surgical services, while the GAM adjusts better in the case of more specialized medical services.

**Conclusions:**

Generalized Additive Models are more flexible than parametric models, providing a better fit in the presence of non-linear relationships and thus allowing more accurate prediction values. The results of this study suggest that AM is a promising technique for the areas of research and application in health economics.

## Background

Production functions are a fundamental component of all economies [1] and the analysis of production functions has been used by economists since the 1930s to study efficiency, and it is one of the econometric methods most commonly used by health economists [8]. In relation to these production models, the concept is defined first, then the model is specified, then the input and output are measured and, finally, the function is used for the measurement of hospital efficiency.

Thus, it is necessary to provide a design of the form and dimensions of the analytical expression where the term production function refers to the physical relationship between the organization of productive resources and the result, in the form of goods or services per unit of time.

One model is commonly used in the estimation of the hospital production function [32]: the Cobb–Douglas model. Cobb–Douglas has been very popular among economists because its calculation is simple. However, theoretical and empirical studies have frequently questioned the validity of the Cobb–Douglas parametric model as a representation of the production of health care services [20].

The Cobb-Douglas production function is applied to the study of production functions, both in a specific business sector and in a sector of the national economy, for the study of the elasticity and substitution of productive factors, which in the classical economy are work, capital, land and production technology. It has been applied to different sectors such as agriculture, as the paper by Rebollar-Rebollar et al. [28] related to milk production or manufacturing, like Smirnov and Wang studying the volume of production in US manufacturing to calculate the elasticity of labor and capital [33] or the same approach for the South African manufacturing production [18]. Likewise, it is used to calculate the cost functions of productive factors in different economic sectors [22, 35].

As well as the calculation of the functions of average productivity and marginal productivity of labor and capital and, in a long-term horizon, the evaluation of the existence of scale economies and scale diseconomies from the perspective of the cost function [12].

Aiyar and Dalgaard [2] find that the specification CD performs reasonably well for certain purposes. Likewise, the ease for their computation is predicated of the said function, based on the possibility of log-linearizing them if a non-linear form is necessary, maintaining the linearity of the parameters for their final computation, this is satisfied by the Cobb-Douglas function. Moreover, if the function belongs to a complete system, the different functions of that system must have the same algebraic form although they have different parameters, which means to predicate uniformity, and this can be said of the Cobb-Douglas function. And, to major, the advantage that the number of parameters is minimized, that is to say, the parsimony, is also predicable of the Cobb-Douglas function [26].

But, on the other side, the Cobb-Douglas Production function is dubbed as a simplistic tool based on restrictive conditions. Apart from criticisms directed at the Cobb-Douglas production function regarding the possible existence of multicollinearity and the assumption of constant returns of scale for production factors, the most serious and extended criticism is the inflexible condition of its functional form. And flexibility is important because it allows information to be provided on critical parameters [10, 26].

The relative lack of flexibility of parametric models has led to the development of nonparametric regression techniques based on the family of generalized additive models (GAMs) [17, 41]. These techniques do not impose a parametric form for the predictors; instead, they assume only that those effects are additive and reasonably smoothed so that they can be estimated using a variety of nonparametric smoothing methods.

The utility of GAM in practical applications has been demonstrated in multiple research areas, with reference, among other disciplines, to biology, medicine [17, 41] and also economics and finance [16].

In different economic sectors, a comparison has been made between the Cobb-Douglas production function and the generalised additive model (GAM) function and to assess whether the latter presents a functional form that better adjusts the parameters to real data than the former one. Thus, Stokes [34] performs an analysis of the monetary balances in relation to the production function, analyzing the Cobb-Douglas, CES, Translogarithmic and GAM functions, concluding that the specification of the Cobb-Douglas log-linear function is not appropriately given that the GAM captures the nonlinearity of the monetary variables in the production function in your example.

Likewise, Collins [6] has obtained the cost functions whose calculation allows evaluating the efficiency at the moment of identifying the optimum in size economies in school districts, concluding that the GAM model allows identifying a relationship between spending and size which is obscured by the assumptions of the Cobb-Douglas model.

However, despite the potential advantages of using GAMs in practice many models have, until now, not been sufficiently explored in health economics problems.

## Objectives

The main objective of this study is the calculation of a new flexible production function for hospitals and hospital clinical and surgical services. Secondly, the main strengths and weaknesses of the different functional forms used in this study, the flexible form and the classic Cobb–Douglas form, are examined; the study then considers how hospital clusters respond to the different models of hospital production functions and, finally, assesses the predictive capacity of additive model (AM) analyses regarding the behaviour of the classic functions, (C) the above for the production functions of clinical services (medical and surgical).

The new GAM model and the classic Cobb–Douglas model are applied to the database of public hospitals of the Galician health service for the 2012–2018 time series.

This study is structured as follows: Section 2 presents information about the Galician public hospital sector, Section 3 summarizes the database under analysis, Section 4 briefly describes the Cobb–Douglas production function and introduces the flexible GAM model, Section 5 calculates the results for the database with these two models, and Section 6 performs a comparison between the two models, evaluating whether the specification of the Cobb–Douglas function fits the hospital production in the period of study. Finally, relevant results are presented regarding the ability of the GAM models to predict the behaviour and adjustment of the classic models, as well as the adjustment of the GAM models themselves for the production functions.

### Galician hospital system

The Spanish national health system was created in 1987, as a development of the Spanish Constitution of 1978. The new model thus established is characterized by its universal coverage, equity criteria and tax financing. In fact, the Spanish Constitution guarantees all citizens the right to health, with the provision of health care services by the health institutions of the public system.

At the beginning of the 1990s, Galicia joined the process of the decentralization of health care resources, which gives the Autonomous Community of Galicia and its government control over health care resources through the creation of its own health service within the national health system in Spain. The public health service in Galicia consists of ten hospitals or hospital complexes, managed by the autonomous organization Service Galego da Saúde (SERGAS).

Some data on the size and characteristics of the hospital sector show that the number of beds in SERGAS hospitals is 7446, and that there are 3917 physicians. In 2008, the number of admissions to Galician hospitals was 248,371, with 2,233,894 overnight stays, 1,431,011 first visits and 1,105,083 emergencies.

## Methods

### Material

#### Inputs and outputs of hospital production

The variables used in the study are the inputs, understood as capital and labour, and the outputs are hospital production.

Hospitals are multiproduct production centres, with a variety of patients being treated with a variety of inputs. There is no consensus on the most accurate measure of the output of hospital production Q, so that researchers have used different indicators to measure it, including the number of discharges, and the number of admissions or overnight stays. However, these measures fail to capture in a convenient way the health care provided by hospitals to patients.

In this investigation, the number of income, standardized by complexity or case-mix, is used as a measure of hospital production, to give a homogeneous unit of production called Unit of Hospital Production (UPH). The UPH is obtained by multiplying the number of income by its complexity obtained from the weights of the DRG [21], thus addressing the need to take into account the complexity of the different hospitals and adjusting to a large extent the production output of each hospital.

Following Ferrier and Valmanis [9], the inputs of the hospitals can be measured as follows: for the capital input, the number of beds is used for each hospital and each year, with these data being obtained from the official hospital statistics. The work input is measured as the number of hospital specialists on the staff of each hospital on December 31 of each year.

#### Data

The data have been collected and organized as panel data from the SERGAS information system, using statistics from the SERGAS hospitals. The data for the hospitals collected for the period 2012–2018 are:

- Total number of beds and services
- Number of medical specialists (total and per service)
- Number of admissions and registrations by Diagnostic Related Groups (DRG) (total and per service)
- Cluster in which each hospital is included
- Number and typology of medical and surgical hospital services per cluster

In Galicia, the hospitals have been classified into three clusters by Reyes [29]. This classification indicates the number of specialties a hospital has, as a reflection of the type of services it provides. For example, Cluster 2 hospitals only provide internal medicine services, general surgery and some basic specialties, while Cluster 3 hospitals provide a considerable range of specialized services. By contrast, Cluster 1 hospitals provide specialized services with high technology medical equipment and highly qualified personnel. In this context, the hospitals with the fewest specialties treat the simplest cases, since, compared with the hospitals in Clusters 1 and 2, they are less well equipped with high technology medical equipment such as computerized tomography or magnetic resonance scanners.

Table 1 shows how the SERGAS Hospital’s distribution according to the different Clusters,

**Table 1:** Hospital’s distribution according to Clusters

Cluster	Hospital
**Cluster 1**	C.H. UNIVERSITARIO JUAN CANALEJO
	C.H. UNIVERSITARIO DE SANTIAGO
	C.H. UNIVERSITARIO DE VIGO
**Cluster 2**	C.H. ARQUITECTO MARCIDE-NOVOA SANTOS
	H. DA COSTA
	H. COMARCAL DE MONFORTE
	H. COMARCAL DE VALDEORRAS
**Cluster 3**	C.H. XERAL-CALDE
	C.H. DE OURENSE
	C.H. DE PONTEVEDRA

while Table 2 contains some descriptive data on the different hospital’s clusters.

**Table 2:** Data of the public hospital sector of Galicia by cluster

	UPHs	Hospital bed supply	Consultants
CLUSTER 1	> 60,000	> 1000	> 500
CLUSTER 2	< 20,000	< 450	< 200
CLUSTER 3	34,000-50,000	550–800	250–500

### Statistical methods

#### Classic model

The Cobb–Douglas function was originally estimated by Charles W. Cobb and Paul H. Douglas [5], although it had already been considered by Knut Wicksell [39] and, according to some authors, by von Thünen [36]. It has the following form:

$$ Q=\alpha {L}^{\beta_1}{K}^{\beta_2} $$

where Q, L and K represent the output, the work factor and the capital factor, respectively, and α, β_1_ and β_2_ are constants.

A problem with this function is the omission of the change in production technology. The need to take this technological change into account was identified by Handsaker and Douglas [15] and Williams [40]. A standard procedure for introducing technological change into the function is to include the time of the series. This allows the function to capture changes in the technology, although it is assumed that this is exogenous to the specification of the function

$$ Q=\alpha (T){L}^{\beta_1}{K}^{\beta_2}, $$

where, *α*(*T*) = *αe*^*φT*^ are constants. φ is a measure of the proportion of the change in the output per period of time, keeping the levels of the inputs constant. This implies that technological change is exogenous.

If we assume that the effects of technological progress are neutral, the form of the Cobb-Douglas production function is simplified as follows:

1 $$ 1\mathrm{n}\ Q={\beta}_0+{\beta}_11\mathrm{n}(L)+{\beta}_21\mathrm{n}(K)+{\beta}_31\mathrm{n}(L)1\mathrm{n}(K)+{\beta}_4T+\varepsilon, $$

where Q represents the aggregate output, T is time, K is fixed capital and L is work. β stands for the parameters of the function.

The Cobb–Douglas model that has been used in this study is:

2 $$ 1\mathrm{n}\; UPHs=1\mathrm{n}\left(\alpha \right)+{\beta}_11\mathrm{n}(FTEs)+{\beta}_21\mathrm{n}(Beds)+{\beta}_31n(FTEs)1\mathrm{n}(Beds)+ Hospital+ Year+\varepsilon, $$

#### The flexible additive model

Flexible generalized additive models (GAMs) are useful as predictors in functional relationships for different groups of data, and avoid the need to establish a specific functional model a priori. These models combine the ability to explore several nonparametric relationships simultaneously, through the flexibility provided by the distributions of generalized linear models (GLM) [23].

In our model, the response variable is continuous. In this case, the generalized additive model is usually referred to in the statistical literature as an additive model. Thus, in the present study we will denote the flexible model by AM. This study proposes the model for both hospital clusters and medical services.

- *Hospitals*

The flexible model considered is AM including the beds–facultative interaction:

3 $$ 1\mathrm{n}\; UPHs={f}_1\left(1\mathrm{n}\; Beds\right)+{f}_2\left(1\mathrm{n}\; FTEs\right)+{f}_3\left(1\mathrm{n}\; Beds,1\mathrm{n}\; FTEs\right)+ Hospital+ Year+\varepsilon, $$

where *f*_1_ and *f*_2_ are unknown smoothed functions of the number of beds (on a logarithmic scale) and the number of practitioners (on a logarithmic scale), respectively, *f*_3_ is an unknown smoothed function representing the possible interaction between the number of beds and the number of physicians (both on a logarithmic scale), and *ε* is the error term, which follows a normal distribution with zero mean. Since the database contains observations for the different medical services for each hospital for the period 2012-2018, the covariates "Hospital" and "Year" have also been included in the model; “Hospital” as a categorical variable [27] and “Year” as a continuous variable [14].

Based on the estimation of the model (3), penalized thin plate splines [41–43] are used to represent the smoothed functions *f*_1_, *f*_2_ and *f*_3_ and the representation of the mixed model of a penalized AM is considered [42]. In this configuration, the smoothed parameters are estimated via REML [19, 37, 38].

b) *Medical Services*

The flexible model considered is:

4 $$ 1\mathrm{n}\; UPHs={f}_1\left(1\mathrm{n}\; Beds\right)+{f}_2\left(1\mathrm{n}\; FTEs\right)+{f}_3\left(1n\; Beds,1\mathrm{n}\; FTEs\right)+ Year+\varepsilon, $$

where *f*_1_ and *f*_2_ are unknown smoothed functions of the number of beds (on a logarithmic scale) and the number of practitioners (on a logarithmic scale), respectively, *f*_3_ is an unknown smoothed function representing the possible interaction between the number of beds and the number of physicians (both on a logarithmic scale) and *ε* is the error term, which follows a normal distribution with zero mean. Since the database contains observations for the period 2012–2018, the covariate “Year” has also been included in the model. For the estimation of the model (4), the same procedure has been followed as for the model (3).

## Results

In this section we present the estimated results for each model, for the whole of SERGAS, for each cluster of hospitals and for selected medical specialties. We assess which specification of the production function (Cobb–Douglas or the flexible additive model) is better adjusted for the public hospital sector of Galicia, in the period 2012–2018.

The models are evaluated based on the AIC (Akaike Information Criterion: [3]) and on the economic interpretation of the changes in output due to changes in the input factors. It should be noted that smaller AIC values reflect a better fit. The value of the corrected *R*^2^ is also presented for each model.

### Hospital clusters

#### The global model

First, the models are estimated for SERGAS globally.

According to Table 3, related to the Global Model, in the two models the variables Beds, Full Time Equivalent consultants (FTEs) and Hospital are all significant (*p* < 0.001 in all cases). In addition, the interaction between beds and facultative is also significant for the flexible AM (*p* < 0.001). However, the variable Year (Year) as an approximation for changes in production technology is not significant (CD *p* = 0.477, AM *p* = 0.690). This result indicates that the change in production technology is neutral in relation to output.

**Table 3:** Results of the Cobb–Douglas (CD) and Flexible AM models for the global SERGAS

Model	Effects	Coefficients	df	***P***-value	***R***^**2**^(× 100%)	AIC
**CD**	Beds	1.030	1.00	< 0.001		
	FTEs	0.147	1.00	0.004		
	Beds X FTEs	−0.053	1.00	< 0.001	78.34	2599.273
	Year	−0.011	1.00	0.477		
	Hospital		9.00	< 0.001		
**AM**	s (Beds)	–	5.44	< 0.001		
	s (FTEs)	–	5.32	< 0.001		
	s (Beds,FTEs)	–	15.00	< 0.001		
	Year	−0.006	11.00	0.6897		
	Hospital		9.00	< 0.001	82.90	2355.801

*AIC* Akaike Information Criterion, *df* degrees of freedom, *s()* smoother using thin plate splines

Regarding the goodness of fit for the models, it is observed that, from the values of *R*^2^ and the AIC, the flexible model presents a better fit than the classic models.

**Fig. 1:**
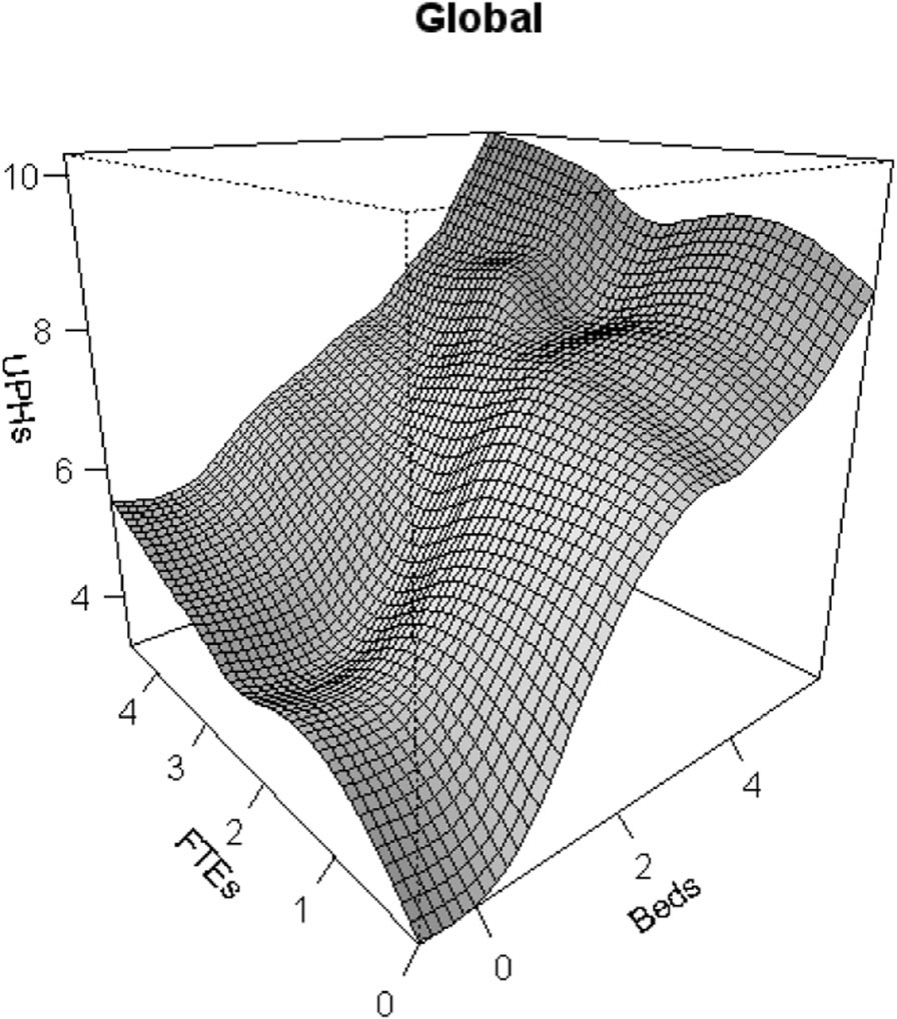
Productivity Growth flexible models: global model (Variables (UPHs, FTEs, Beds) are expressed in Logarithm scale)

Figure 1, related to the Global model, shows three lines. The upper line represents the change in productivity according to variations in the input. The lower right line represents the variations in the capital factor (Beds) while the lower left line shows the variations in the work factor (FTEs).

According to the upper line, Fig. 1 shows a rapid increase in productivity. This increase is determined by the trend of the capital factor variable while the contribution of the work factor to the increase in productivity is quite modest.

#### Estimates of the models for the hospital clusters

The results for Cluster 1 are presented in Table 4. The first noteworthy aspect for the estimated variables is that, unlike the results of the previous section, no statistical significance was found for the Beds variable with either the flexible model or the classic model. However, the flexible model presents statistical significance for the facultative variable (FTEs), while the classic model do not. In addition, the flexible model is the only one to capture statistical significance for the interaction between beds and physicians. None of the models finds statistical significance for the change in production technology or for the Hospital variable.

**Table 4:** Results of the Cobb–Douglas (CD) and Flexible AM models for Cluster 1

Model	Effects	Coefficients	df	***P***-value	***R***^**2**^(× 100%)	AIC
**CD**	Beds	0.684	1.00	< 0.001		
	FTEs	−0.128	1.00	0.205		
	Beds X FTEs	0.051	1.00	0.098	63.54	1072.302
	Year	−0.010	1.00	0.683		
	Hospital		2.00	0.848		
**AM**	s (Beds)	–	4.38	0.193		
	s (FTEs)	–	5.42	0.018		
	s (Beds,FTEs)	–	15.16	0.041	78.50	875.337
	Year	0.015	1.00	0.453		
	Hospital		2.00	0.970		

*AIC* Akaike Information Criterion, *df* degrees of freedom, *s()* smoother using thin plate splines

Looking at the AIC and *R*^2^ values, we can observe greater explanatory power for the flexible model than the classic models.

**Fig. 2:**
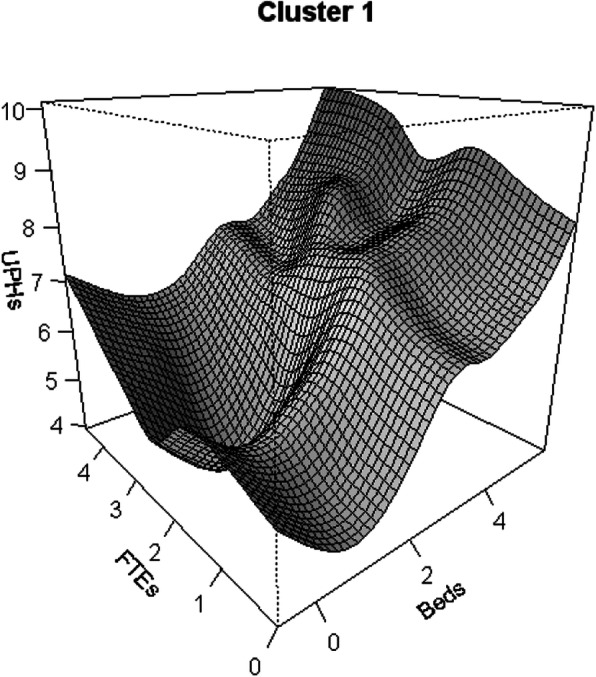
Productivity Growth flexible models: hospital cluster 1 (Variables (UPHs, FTEs, Beds) are expressed in Logarithm scale)

The evolution of productivity in the output for Cluster 1, based on the behaviour of the two production factors evaluated in this study, is shown in Fig. 2. The changes in productivity show that, while the facultative variable (FTEs) gives continuous increases followed by a decrease, the capital variable (Beds) gives sustained increases throughout its curve. On the other hand, the product seems to have decreased return to scale.

Table 5 contains the results for Cluster 2. It is interesting to note that the variable factor of capital, represented by the number of beds (Beds), is statistically significant for the two models, while the work production factor (FTEs) is not significant for the AM model (0.638). In addition, the effect of the interaction between production factors is also captured by the Cobb-Douglas and AM models. The AM model, unlike the classic model, even shows itself capable of capturing the effects that changes in production technology, identified through the time series, produce in the output. The significant statistical effect in the two models of the hospital variable shows some variability in the size of the hospitals belonging to Cluster 2.

**Table 5:** Results of the Cobb–Douglas (CD) and Flexible AM Cluster 2 models

Model	Effects	Coefficients	df	***P***-value	***R***^**2**^(×100%)	AIC
**CD**	Beds	1.167	1.00	< 0.001		
	FTEs	0.262	1.00	0.015		
	Beds X FTEs	− 0.107	1.00	0.002	78.32	728.013
	Year	−0.049	1.00	0.140		
	Hospital		3.00	< 0.001		
**AM**	s (Beds)	–	5.66	< 0.001		
	s (FTEs)	–	3.38	0.638		
	s (Beds,FTEs)	–	10.40	< 0.001	85.30	627.366
	Year	−0.063	11.00	0.022		
	Hospital		3.00	< 0.001		

*AIC* Akaike Information Criterion, *df* degrees of freedom, *s()* smoother using thin plate splines

As in the previous results, both the AIC and the *R*^2^ show that the AM model is a better fit than the classic model.

**Fig. 3:**
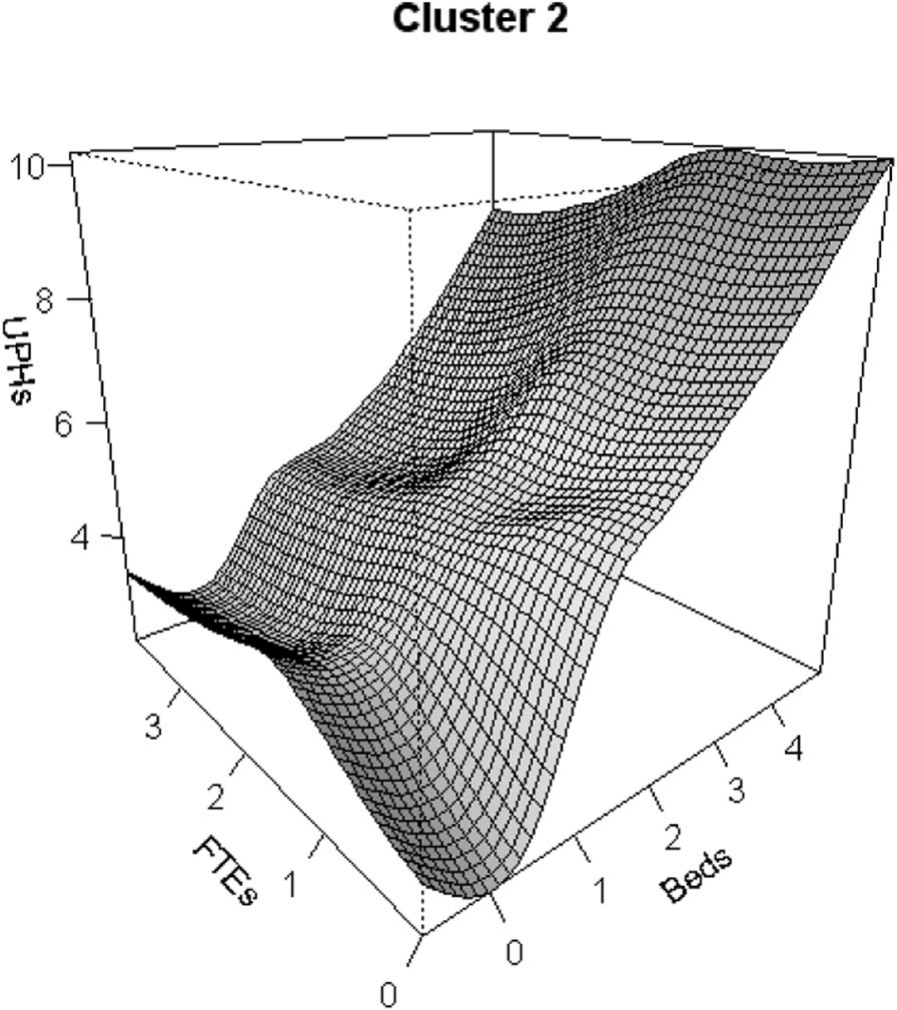
Productivity Growth flexible models: hospital cluster 2 (Variables (UPHs, FTEs, Beds) are expressed in Logarithm scale)

Figure 3 shows, for Cluster 2, an almost linear increase in productivity for increases in the capital factor (Beds), while increases in the labour factor (FTEs) have a lower contribution to the increase in productivity.

The estimated results for Cluster 3 are presented in Table 6. Statistically significant values are shown for the variable Beds (Beds) in the two models, while the optional variable (FTEs), Hospital and change in technology present statistical significance only in the AM model. Likewise, only the AM model shows statistical significance for the interaction between work and capital.

**Table 6:** Results of the Cobb–Douglas (CD) and Flexible AM Cluster 3 models

Model	Effects	Coefficients	df	***P***-value	***R***^**2**^(× 100%)	AIC
**CD**	Beds	0.904	1.00	< 0.001		
	FTEs	−0.057	1.00	0.545		
	Beds X FTEs	−0.011	1.00	0.772	77.96	
	Year	0.023	1.00	0.363		777.311
	Hospital		2.00	0.418		
**AM**	s (Beds)	–	4.20	< 0.001		
	s (FTEs)	–	6.10	0.037		
	s (Beds,FTEs)	–	16.41	0.022	86.50	619.218
	Year	0.021	1.00	0.293		
	Hospital		2.00	0.814		

*AIC* Akaike Information Criterion, *df* degrees of freedom, *s()* smoother using thin plate splines

The goodness of the fit of the models, measured by the *R*^2^ and the AIC, shows advantages for the flexible AM model over the classic model.

**Fig. 4:**
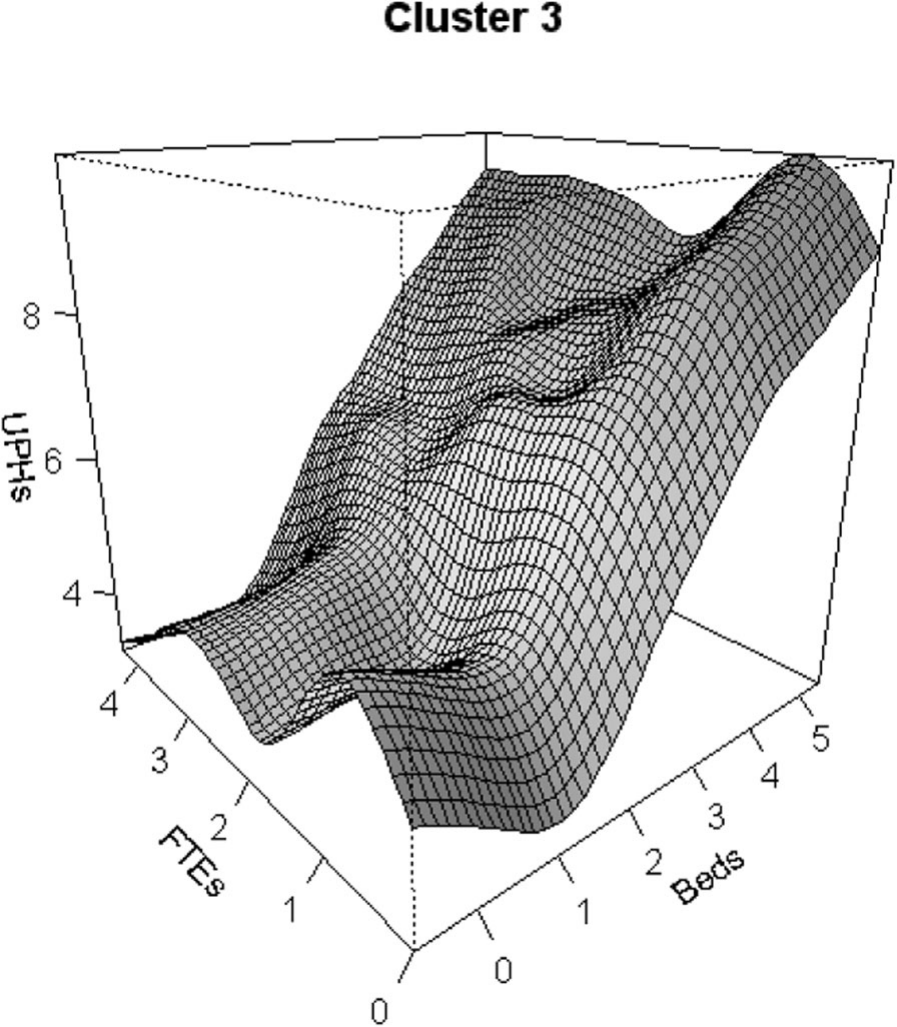
Productivity Growth flexible models: hospital cluster 3 (Variables (UPHs, FTEs, Beds) are expressed in Logarithm scale)

Figure 4 presents, for Cluster 3, the gains in productivity arising from additional resources in the capital factor, reflected by a linear relationship between output and the capital factor. However, the trend for the labour variable is represented by a quadratic curve that shows gains and reductions in production as a consequence of increases in that factor.

#### Clinical and Surgical services

Table 7 shows the results for the specialty of General Surgery for the two models. For the classic model, both factors, capital (Beds) and labour (FTEs), are statistically significant although the variables of technological change and the interaction between the two productive factors are not significant. On the other hand, the flexible AM ​​model shows statistical significance for the work variable and its interaction with capital, but the capital factor and technological change variables are not significant.

**Table 7:** Results of the Cobb–Douglas (CD) and Flexible AM General Surgery models

Model	Effects	Coefficients	df	***P***-value	***R***^**2**^(×100%)	AIC
**CD**	Beds	0.645	1.00	< 0.001		
	FTEs	0.656	1.00	< 0.001		
	Beds X FTEs	− 0.054	1.00	0.364	96.30	−46.109
	Year	−0.002	1.00	0.789		
**AM**	s (Beds)	–	1.00	0.330		
	s (FTEs)	–	1.00	< 0.001		
	s (Beds,FTEs)	–	12.88	< 0.001	98.30	−46.246
	Year	−0.009	1.00	0.441		

*AIC* Akaike Information Criterion, *df* degrees of freedom, *s()* smoother using thin plate splines

The results for the AM model show a better fit than the Cobb-Douglas model. This is an excellent example of the ability of the flexible AM ​​model as an explorer of the goodness of fit of other models. In this case, we are shown that we can trust the calculations and results offered by the Cobb–Douglas model.

**Fig. 5:**
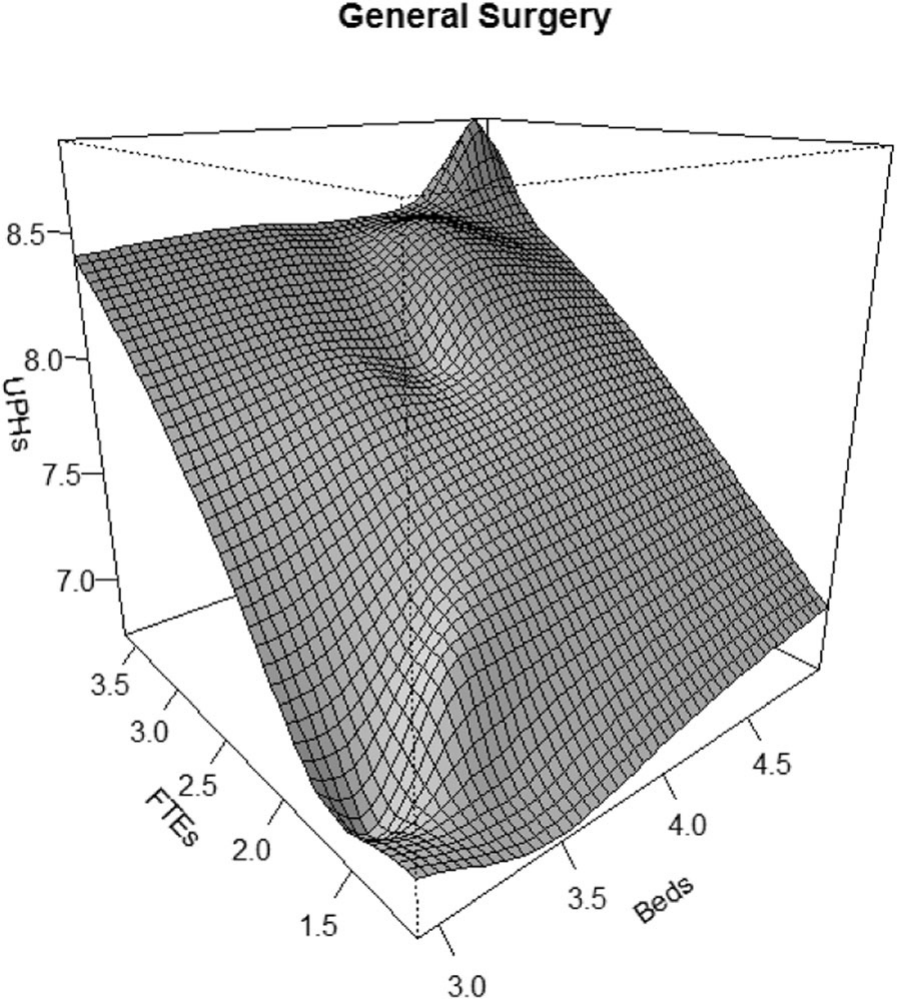
Productivity Growth flexible model: General Surgery services. (Variables (UPHs, FTEs, Beds) are expressed in Logarithm scale)

Figure 5 presents, for the specialty of General Surgery, poor gains in output as a consequence of a continuous and sustained increase in the labour factor, while the capital factor shows small increases in the product as a consequence of continued increases in the factor.

Considering Table 8, the results for the specialty of Internal Medicine show statistically significant values for the variable of capital in the two models, but do not present significant values for the variables of work or technological change in any of the models. In the same way, the variable of interaction between the two productive factors is significant in the flexible AM model but not in the Cobb-Douglas.

**Table 8:** Results of the Cobb–Douglas (CD) and Flexible AM ​​Internal Medicine models

Model	Effects	Coefficients	df	***P***-value	***R***^**2**^(×100%)	AIC
**CD**	Beds	0.831	1.00	< 0.001		
	FTEs	0.056	1.00	0.784		
	Beds X FTEs	0.016	1.00	0.715	95.80	−15.939
	Year	0.012	1.00	0.530		
**AM**	s (Beds)	–	1.00	< 0.001		
	s (FTEs)	–	1.00	0.262		
	s (Beds,FTEs)	–	12.16.00	< 0.001	97.90	−13.113
	Year	0.028	1.00	0.091		

*AIC* Akaike Information Criterion, *df* degrees of freedom, *s()* smoother using thin plate splines

Following the AIC values, the Cobb–Douglas model seems to present a better fit than the flexible model, although for the values of the *R*^2^ the flexible model AM would present the best fit.

**Fig. 6:**
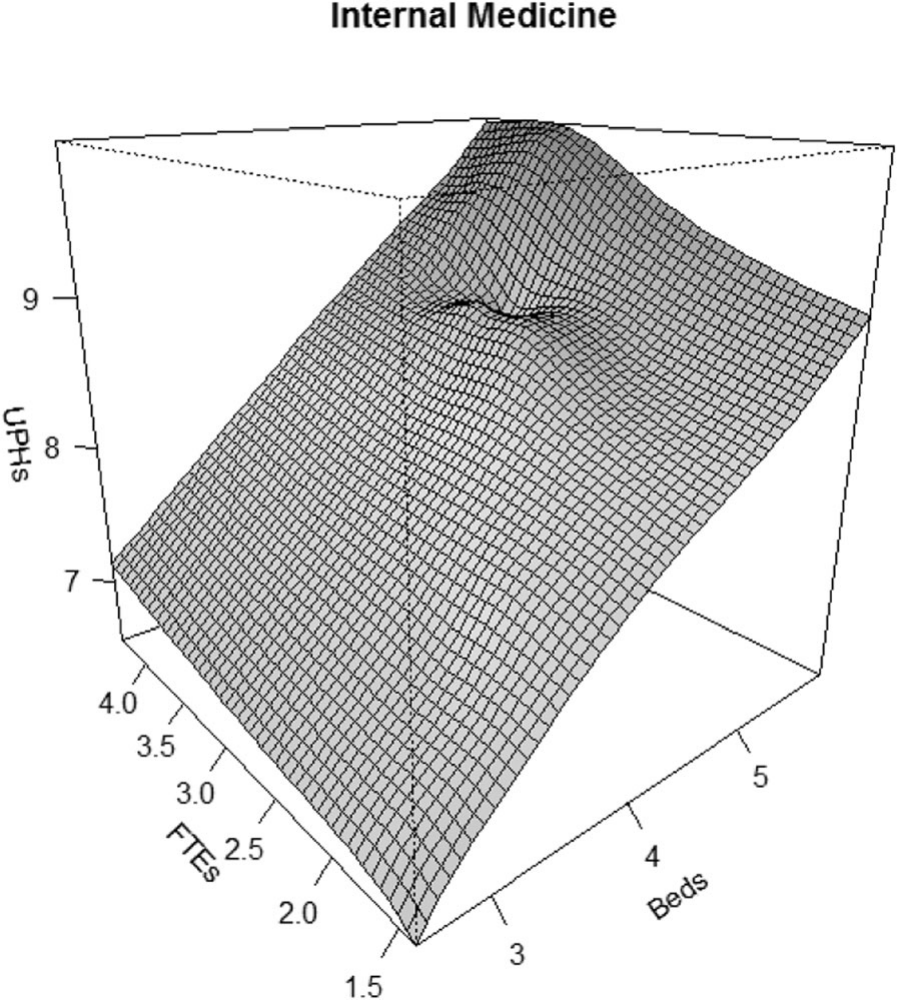
Productivity Growth flexible model: Internal Medicine services. (Variables (UPHs, FTEs, Beds) are expressed in Logarithm scale)

The evolution in productivity for the specialty of Internal Medicine is presented in Fig. 6, which shows gains in production as a linear relationship between the product and the variable capital (Beds) with little influence of the increases in the variable work (FTEs).

## Discussion

This paper studies the potential of additive flexible models as an instrument for calculating the production functions of hospitals and of medical and surgical specialties. The results obtained with the flexible model AM have been compared with those of the function more used in the field of health care; the Cobb–Douglas function.

In this work, the variable Year, as a representation of the changes in production technology, does not show any statistical significance for the global model, which would indicate that technological changes are neutral in relation to output. The use of the variable time as an approach to technological change has been used in multiple studies, but following Blank [4], innovations are slowly spreading to reach all hospitals, thus we can find different hospitals that operate with different technologies, coinciding in the same period of time however, in the present study the variable of technology (Year) is significant for Cluster 2. Likewise, in the work of Mohammadi and Meskarpour-Amiri [14], they also use the year variable as a proxy for production technology in calculating the production functions of a sample of Iranian public hospitals, and it is significant.

Another example of the above is the work of Meyer [25] on the application of an economic function of production in hospitals with different levels of integration in their information systems. The study included 17 public hospitals of the Public Assistance of the Paris Region that were followed up in the period 1998–2005. Author used Cobb–Douglas production function, where the output was correlated with the three stablished production factors: capital factor, labour factor, and information technology, as a proxy of production technology. The calculations done indicate that the higher the level of integration of the information system, the greater is its positive influence on the level of hospital production, what shows the influence of the production technology in the output of the function.

In addition, Cruz-Gomes et al. [7] in their study on 142 Portuguese hospitals, find the variable production technology significant to a greater extent for the activity of surgery services, while our study obtained relevant significance to the proxy variable of production technology only for medical specialty services.

The results related to the capital factor (Bed) in our study present different tendencies according to the hospital clusters, and the services should be analyzed in relation to other studies that address this issue. In this respect, attending the study by Giancotti et al. [11], who performed a peer review study looking for what could be the optimal productive size of hospitals; reporting evidence of economies of scale for hospital’s size between 200 and 300 beds and diseconomies of scale for those hospitals below 200 beds and above 600 beds.

In fact, in the present study, the factor with the highest coefficient and, therefore, the highest elasticity relative to final production, for all hospitals and for each of the clusters, is the capital factor, higher than the work factor. In the same way, following Rezapoor et al. [30], some results of their study were that among the production variables that affect the industry studied by the authors, the contributions of doctors, beds and other personnel had the positive effect; being the most positive effect belonged to the capital factor or active beds and the minimum impact was related to doctors while nurses as input had a negative impact.

In the same sense, the work by Mehraban et al. [24], concludes that the hospitals of the selected sample belonging to the Iranian Social Security are capital intensive, with incremental returns of scale, as in our work.

On the contrary, the aforementioned study carried out by Mohammadi and Meskarpour-Amiri [14], revealed that for the Iranian public hospitals, the elasticity of the level of hospital service in terms of labour factor is greater than the capital factor, said beds. Likewise, the work carried out by Hamidi [13] shows a greater contribution of the work factor, in particular the doctors, over the capital factor. Furthermore, it shows decreasing returns to scale, as does our study for all hospitals. Authors also obtain a greater elasticity for the coefficients of the work factor in the Cruz-Gomes [7] study, this elasticity being greater for surgical services than for medical services, the latter finding coinciding with our study.

And, related to the study by Romley and Sood [31], in terms of public policy, their finding that as the returns to hospital care are positive this could suggest that reductions in hospital spending could have adverse effects on patient outcomes.

In the results of our study model for specialties, we have observed the possible existence of returns of scale in certain services, but not in all. In that sense, for Cruz-Gomes et al. [7], in their study, they also obtain returns of scale for surgery services, while our study observes decreasing returns of scale.

Coincident with the best fit of the GAM non-parametric model compared to the Cobb-Douglas model, it coincides with the results of Cruz-Gomes [7] that also find a better fit of its flexible model compared to the CD. Likewise, both the study cited by Hamidi [13] and Mohammadi and Meskarpour-Amiri [14], conclude in their studies that the translog model fits better than the Cobb-Douglas.

Finally, and in relation to the model analyzed in this study, we must consider that an additive structure allows the factors to be added in indexes. Thus, the elasticities or substitution ratios can be derived directly and, in addition, the GAMs allow the calculation of the second derivative. In summary, GAMs are easily interpretable in the field of health economics. In addition, the additive models allow us to evaluate the behaviour of the linear models and therefore to evaluate their application and behaviour.

## Conclusions

The results of this study suggest that the Flexible Additive Model is a promising methodology for the study applied in the field of health economics, due to its better fit for the prediction of the behaviour of the variables of hospital production functions. In addition, this methodology can be extended to studies on cost, demand and utility functions in the field of health economics.

The study faces a double comparison between the Cobb-Douglas parametric model and the flexible GAM model: on the one hand, to evaluate which model presents a better fit to the real data and, on the other, to evaluate the elasticities of the production factors and their returns of scale. It is unquestionable that, given a better adjustment of the function to reality, the possibility of adjusting investments in the different productive factors to increase their productivity, with a greater degree of effectiveness in decisions, increases the probability of achieving improvements.

The flexibility of additive models offers interesting advantages for research in other areas of health economics. Even when the data handled are susceptible to more traditional techniques, AMs can, as in the present study, provide useful verification of the validity of less sophisticated methods, such as Cobb-Douglas model.

## Data Availability

Data is available upon request to authors

## Abbreviations

AM: Additive Model
DRG: Diagnostic Related Groups
FTE: Full Time Equivalent consultants
GAM: Generalized Additive Model
GLM: Generalized Linear Model
SERGAS: Servicio Galego da Saude (Galician’s Regional Health Service)
UPH: Unit of Hospital Production
